# Therapeutic vaccines targeting HPV epitopes in human papillomavirus – positive oropharyngeal cancer: a critical review

**DOI:** 10.3389/fimmu.2025.1727804

**Published:** 2026-01-19

**Authors:** Giovanna Rossi, Francesco Carlo Tartaglia, Nerina Denaro, Michele Ghidini, Funda Goker, Ornella Garrone, Aldo Bruno Giannì, Massimo Del Fabbro, Gianluca Martino Tartaglia, Paolo Bossi, Alberto Paderno

**Affiliations:** 1 Department of Biomedical, Surgical and Dental Sciences, University of Milan, Milan, Italy; 2 Department of Biomedical Sciences, Humanitas University, Milan, Italy; 3 Medical Oncology, Fondazione Istituto di Ricovero e Cura a Carattere Scientifico (IRCCS) Ca’ Granda, Ospedale Maggiore Policlinico, Milan, Italy; 4 Unità Operativa Complessa (UOC) Maxillo-Facial Surgery and Dentistry, Fondazione IRCCS Ca’ Granda, Ospedale Maggiore Policlinico, Milan, Italy; 5 Department of Oral and Maxillofacial Surgery, Faculty of Dentistry, Istanbul Aydın University, Istanbul, Türkiye; 6 Medical Oncology and Hematology Unit, IRCCS Humanitas Research Hospital, Rozzano, Milan, Italy; 7 Otorhinolaryngology Unit, IRCCS Humanitas Research Hospital, Rozzano, Milan, Italy

**Keywords:** cancer immunotherapy, clinical trials results, combination therapy, HPV oropharyngeal squamous cell carcinoma (OPSCC), immuno-oncology, therapeutic cancer vaccine

## Abstract

Human papillomavirus-positive (HPV+) oropharyngeal squamous cell carcinoma (OPSCC) is a peculiar entity, with distinct patient, tumor, and biological characteristics, and a different prognosis compared to HPV-negative (HPV-) OPSCC. Due to the rising incidence, there is a need to develop novel therapeutic approaches, especially considering the long-term morbidities of traditional treatments such as surgery and chemoradiotherapy. In this regard, therapeutic vaccines targeting HPV epitopes have been put at the forefront of the immunotherapeutic strategies for HPV+ OPSCC. Multiple clinical trials are investigating their efficacy and safety in the advanced setting, more frequently as a combination therapy. As for the early setting, HPV therapeutic vaccines could represent a strategy to further deepen responses and to facilitate de-escalation of standard treatment. This review aims to provide the clinician with useful and up-to-date information on the current advances in this field. To that end, we will provide the results of the main ongoing/completed clinical trials including patients with OPSCC, focusing on immunogenicity and clinical benefit, in both early and advanced setting. We will also map the challenges and limitations in this area to guide future research.

## 1 Introduction

Human papillomavirus-positive (HPV+) oropharyngeal squamous cell carcinoma (OPSCC) is a peculiar entity, with distinct patient, tumor and biological characteristics, and a different prognosis compared to HPV-negative (HPV-) OPSCC (1). In fact, HPV+ OPSCC is diagnosed more often at an early stage and with nodal metastasis (2). The prevalence of HPV+ OPSCC is higher in non-smokers and non-alcohol drinkers (3, 4) and sexual behavior represents an established risk factor (4). Alterations in gene encoding DNA damage response proteins, as well as in immune-related genes, are found more frequently in HPV+ OPSCC than in HPV- OPSCC (5). The better outcomes observed in patients with HPV+ OPSCC led the American Joint Committee on Cancer (AJCC) to recommend a classification based on HPV status in the 8^th^ Edition of the AJCC staging system, in order to improve risk stratification and prognostic accuracy (6). However, the incidence of HPV+ OPSCC is rising in high-income countries (7), both in smokers and non-smokers (8), and it is expected to rise in the coming decades until the benefit of prophylactic HPV vaccination will emerge. Although HPV+ OPSCC is more chemo/radiosensitive, the sensitivity is reduced in smokers with HPV+ OPSCC (9), thus increasing the risk of cancer progression and death (3).

For these reasons, there is a need to develop novel therapeutic approaches, particularly due to the long-term morbidities associated with traditional treatments and the failure of the first generation of randomized chemotherapy deintensification trials (10).

In this regard, immunotherapeutic modalities have revolutionized the treatment of OPSCC. On the one hand, immune checkpoint inhibitors (ICI) demonstrated remarkable efficacy in the advanced setting (11, 12), and have been approved as a first-line treatment for recurrent and/or metastatic (R/M) head and neck squamous cell carcinoma (HNSCC) as well as platinum-resistant R/M HNSCC, including OPSCC. On the other hand, therapeutic vaccines have been brought to the forefront of immunotherapeutic strategies for HPV+ OPSCC.

**Figure 1:**
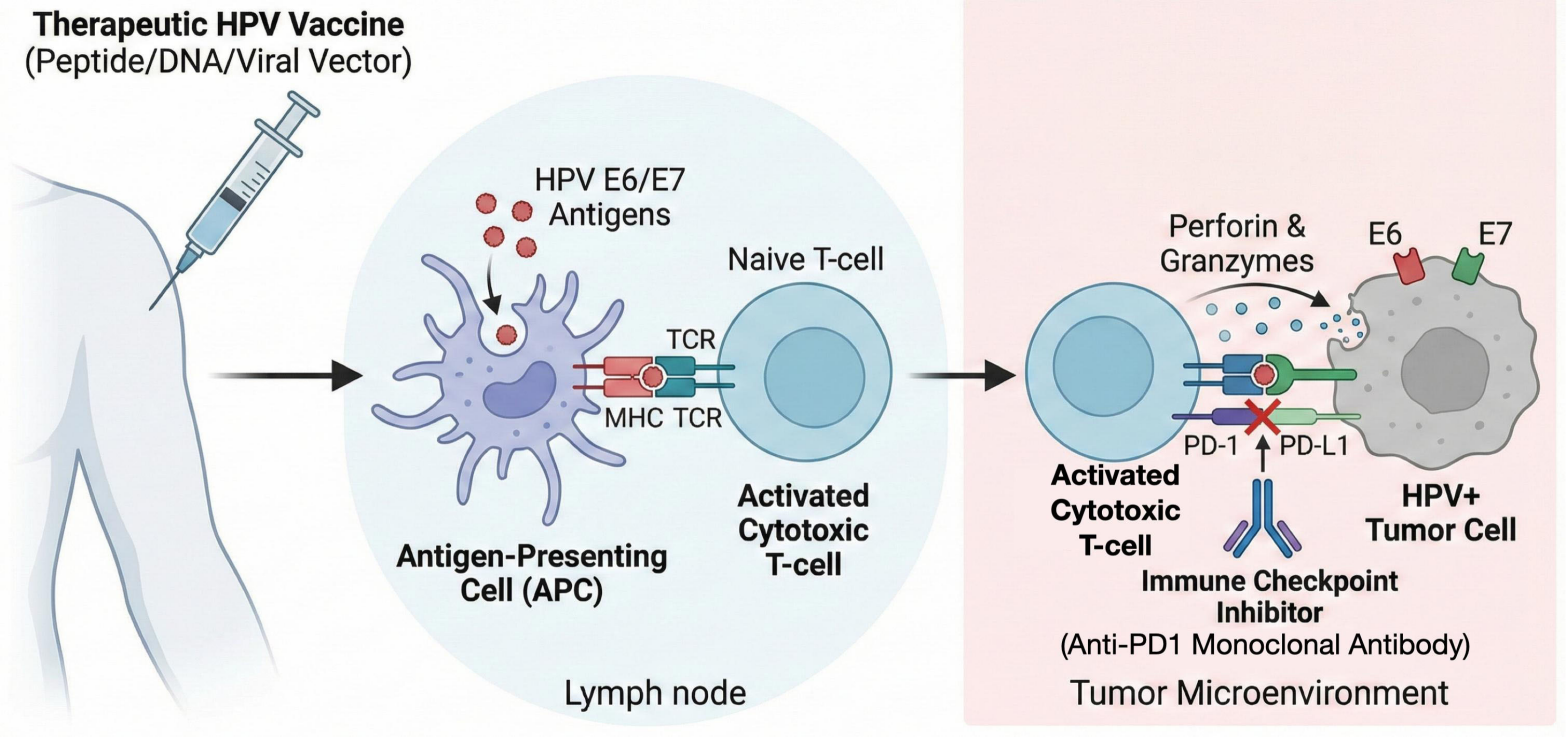
Mechanism of action of therapeutic HPV vaccines and synergic action of immune checkpoint inhibitors. HPV therapeutic vaccines elicit the identification of malignant cells by exposing effective cancer antigens, inducing an endogenous T-cell response, and interrupting immune tolerance. Immune checkpoint inhibitors block checkpoint proteins on the surface of activated cytotoxic T cells from binding with their partner proteins on HPV+ tumor cells. This prevents the “off” signal from being sent to T cells, allowing them to kill cancer cells.

A therapeutic cancer vaccine aims to elicit the identification of malignant cells by exposing effective cancer antigens, inducing an endogenous T-cell response, and interrupting immune tolerance (4) (**Figure 1**). In HPV+ OPSCC, cancer cells infected with HPV generate non-self-antigens, that may become potential targets for a vaccination strategy (13).

**Figure 2:**
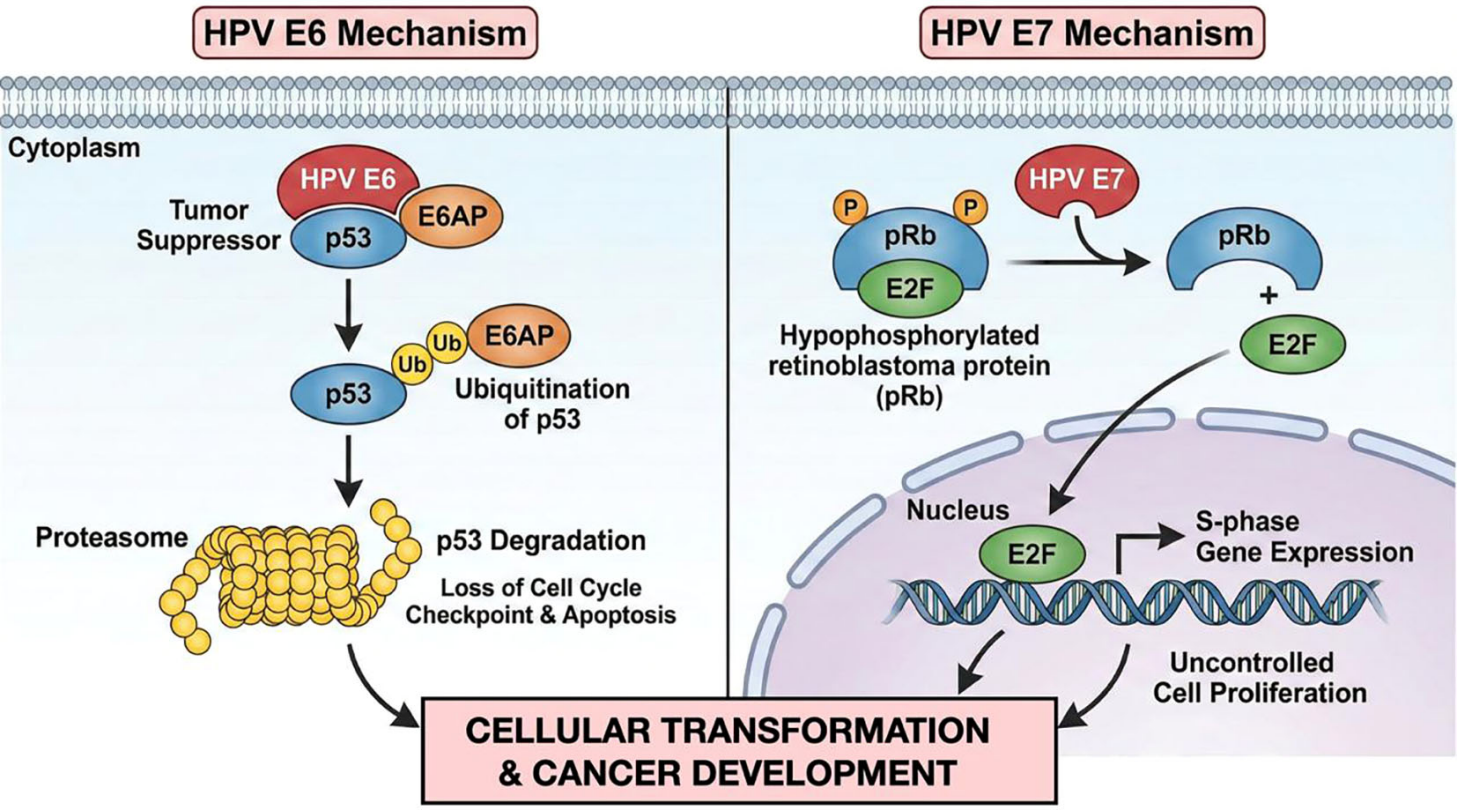
Mechanism of action of the HPV E6 and E7 oncoproteines. HPV E6 and E7 oncoproteins induce tumor development by inactivating key tumor suppressors: E6 targets p53 for degradation, blocking cell cycle arrest and apoptosis, while E7 binds and inactivates pRb, releasing E2F to force cells into the S-phase (DNA synthesis) of the cell cycle, causing uncontrolled proliferation and genomic instability. Their synergistic action generates a cellular environment in which damaged cells are forced to replicate and accumulate mutations, thus promoting cancer development.

The majority of HPV therapeutic vaccines currently in clinical testing target E6 and E7 oncoproteins, which represent ideal targets, as they are constitutively expressed, restricted to cancer cells, and present at high levels across patients. Moreover, E6 and E7 are essential for both the initiation and maintenance of malignancy (13) (**Figure 2**).

This review aims to provide clinicians with useful and up-to-date information on the current advances in HPV epitope-based therapeutic vaccines in patients with OPSCC. To that end, three databases (PubMed, Embase, and Scopus) and a clinical trial registry (clinicaltrials.gov) were searched up to August 2025. Moreover, congress proceedings related to the abstracts presented at the American Society of Clinical Oncology (ASCO), European Society for Medical Oncology (ESMO), and ESMO Immuno-Oncology Congresses in the last 10 years were consulted. Full-text papers were also screened for additional hand-search references.

Here, we will provide the results of the main ongoing/completed clinical trials, including patients with OPSCC, focusing on immunogenicity and clinical benefit, in both early and advanced settings. We will also offer a critical view of the challenges and the limitations in this area, in order to guide future research.

The basic principles of therapeutic vaccines, or the current state of art on non-HPV epitope-based interventions in the broader context of HNSCC, will not be covered, since these topics were extensively presented in other review articles (13, 14).

## 2 HPV therapeutic vaccines for HPV+ OPSCC in the advanced setting

The development of therapeutic vaccines targeting HPV-related antigens in patients with R/M OPSCC is a rapidly evolving field, and multiple clinical trials are investigating their efficacy and safety in this setting, often as a combination therapy.

### 2.1 Completed or active but not recruiting clinical trials

#### 2.1.1 Peltopepimut-S (ISA101b)

In the R/M setting, the synthetic long peptide (SLP)-based vaccine ISA101b, containing 12 SLPs derived from the E6 and E7 oncogenic proteins, was evaluated in few clinical trials enrolling patients with OPSCC only. In the phase II trial NCT03669718 (15), patients with R/M HPV-16+ OPSCC first- and second-line ICI naive, were randomized to receive ISA101b plus the anti-programmed cell death protein 1 (PD1) cemiplimab or placebo plus cemiplimab. ISA101b (100 µg/peptide) or matching placebo were injected subcutaneously following a 3-dose schedule (days 1, 29 and 50), while cemiplimab (350 mg) was administered intravenously on day 8, and then every 3 weeks up to 24 months after treatment start or until disease progression or withdrawal for other reasons. A total of 198 patients (mean age 62.8 ± 9.6 years) received ≥1 dose of study drug: 173 (87.4%) males, and 25 (12.6%) females; 110 (55.6%) were treated in the first line, and 74 (37.4%) in the second line, while for 14 patients (7%) this data was unknown. Considering the full analysis set, in the ISA101b arm (91 patients), objective response rate (ORR) as per Response Evaluation Criteria in Solid Tumors (RECIST) 1.1 was 25.3% compared to 22.9% in the control arm (96 patients), *p=*0.590. However, in a subset analysis with a limited number of patients, the ones with a combined positive score (CPS) ≥20 treated with cemiplimab and a full course of vaccine had a significantly better ORR (61.9% *versus* 28.0%, *p=0.026*) and overall survival (OS) (median OS (mOS) 95% confidence interval (CI) not reached (NR) (28.1, -) *versus* 23.3 (11.9-30.1) months, *p=0.0232*, in the per protocol set). Conversely, patients with lower CPS did not benefit. Toxicity was comparable between the 2 arms.

In the single-arm phase II study NCT04398524 (16, 17), patients with R/M HPV-16+ OPSCC that progressed on or soon after anti-PD1 therapy were treated with peltopepimut-S (subcutaneously 100 mg/peptide) following a 3-dose schedule (days 1, 29 and 50) plus cemiplimab (intravenously 350 mg as a 3-weekly regimen) until disease progression. Patients still on the study after 6 months were offered a single vaccine booster injection. After a median follow-up of 40.9 (range 1.6-138.7) weeks (17), among the 64 patients (mean age 61.5 ± 10.6 years; 57 [89.1%] males) included in the efficacy analysis, the ORR as per RECIST 1.1 was 6.3% (4 partial responses (PR)). The clinical benefit rate (CBR), consisting of partial response (PR) and stable disease (SD), was 56.3% (36 patients). Regarding the survival outcomes, mOS was 11.3 (0.3-29.7) months, and median progression-free survival (mPFS) was 3.6 months (95% CI: 1.8-4.7). The combination of peltopepimut-S and cemiplimab was well tolerated, with 4 grade 3 adverse events (AEs): one local toxicity and 3 immunotoxicities.

The single-arm phase II trial NCT02426892 enrolled patients with HPV-16+ cancers and was not restricted to patients with OPSCC. However, 22 (20 males, 2 females) out of 24 participants had OPSCC and data could be extracted for this population (18). ISA101b, 100 μg/peptide, was administered subcutaneously on days 1, 22, and 50, and nivolumab, 3 mg/kg, was given intravenously every 2 weeks beginning day 8 for up to 1 year. Eight patients, all with OPSCC, achieved a response (per RECIST 1.1): 2 complete responses (CR) and 6 PR, for an ORR of 33% (90% CI, 19%-50%). The median duration of response was 10.3 months (95% CI, 10.3 months to inestimable), with 5/8 responses ongoing at the time of analysis. PFS and OS in the 22 patients with OPSCC were identical to those in the overall population: mPFS was 2.7 months (95% CI, 2.5-9.4 months) and mOS was 17.5 months (95% CI, 17.5 to inestimable), with a median follow-up duration of 12.2 months. After vaccination, a variable increased number of HPV-specific T cells by Enzyme-Linked ImmunoSpot (ELISpot) assay was observed in both responders (exclusively patients with OPSCC) and non-responders. The immune response did not correlate with any efficacy endpoints, suggesting that local factors in the tumor environment have a greater influence on vaccine effect. Notably, the OPSCC population was heterogeneous, comprising patients with different lines of treatment received and a consequently heterogeneous immune responsivity. The long-term results of this trial (19), with a median follow-up time of 46.5 months (95% CI, 46.0 to NR), showed a median duration of response of 11.2 months (95% CI, 8.51 to NR). Among the 8 patients with OPSCC who previously responded, 5 progressed, 2 were free of recurrence at 46 and 47 months, and one died without recurrence for a non-cancer-related cause. At this stage, the tumor microenvironment of HPV+ tumors from 17 evaluable baseline biopsies, 6 (35%) of which from responders (all patients with OPSCC) and 11 (65%) from non-responders, was evaluated. The 2 patients with OPSCC who achieved a CR had the highest degree of Cluster of Differentiation (CD) 8+ T cells. Moreover, gene expression analysis revealed differential regulation of 357 genes (≥1.25 fold) in non-responders *versus* responders (p<0.05).

These promising results led the Food and Drug Administration (FDA) to grant the Fast Track Designation to ISA101b for its use in R/M HPV-16+ OPSCC (20).

#### 2.1.2 AMV002

AMV002 is a DNA-based therapeutic vaccine, consisting of plasmids encoding 2 variants of an HPV16 E6 and E7 fusion protein. This compound was evaluated in association with durvalumab in 12 male patients (median age 64) with R/M HPV+ OPSCC enrolled in the phase Ib study ACTRN12620000406909 (21). AMV002–1 mg was administered intradermally in 3 doses, every 4 weeks. Durvalumab was initially administered using a weight-based (10 mg/kg) dose by intravenous infusion on days 7 and 28, followed on day 56 with a fixed dose of 1,500 mg every 28 days for 12 months. Nine out of 11 evaluated patients (82%) exhibited increased E6 and/or E7-specific T-cell responses compared to baseline at one or more time points (days 7, 35, 63, and 84 after the first dose of AMV002), as assessed through the ELISpot assay. At week 16, ORR, measured per immune RECIST (iRECIST), was 8% (n=1) and disease control rate (DCR) was 17% (n=2). Ten patients had progressive disease (PD), 1 SD and 1 had PR at week 16 that became CR at end of study visit. At a median follow-up of 25.6 (20.0-26.6) months, there was one long-term CR while all other participants developed PD. All vaccine-related AEs were mild in severity, with the most common being local grade 1 toxicity. It is noteworthy that the study population was heterogeneous with regards to Programmed Death-Ligand 1 (PD-L1) status, since 3 patients were assessed as PD-L1- and only 2 patients had PD-L1 CPS ≥20, considered favorable for benefit from ICI monotherapy. Moreover, two-thirds of the patient population had received prior systemic therapy.

Some trials on HPV epitope-based therapeutic vaccines open to recruit patients with HPV+ HNSCC or other HPV+ solid tumors also enrolled patients with HPV+ OPSCC, and in certain studies, they represent the majority. In order to provide a comprehensive overview of the topic, we report here the results, specifying their proportion. When analyzing the data, it should be taken into account that they referred to a population that includes some patients, very few or a larger number, with HNSCC from sites other than oropharynx, or with other HPV+ cancers, such as anal or cervical cancer.

#### 2.1.3 MEDI0457 (INO-3112)

MEDI0457 (previously called INO-3112) is a DNA vaccine consisting of 3 plasmids expressing HPV 16/18 E6 and E7 oncoproteins and interleukin (IL)-12 as an adjuvant to increase immune response. MEDI0457 was evaluated in association with durvalumab in the phase Ib/IIa, single-arm study NCT03162224, which enrolled patients with HPV+ R/M HNSCC (22, 23). 7 mg of vaccine were administered by intramuscular injection, immediately followed by electroporation with the CELLECTRA device, on day 1 of weeks 1, 3, 7, and 12, then every 8 weeks. Durvalumab 1,500 mg was administered intravenously every 4 weeks starting from week 4. The treatment was continued until confirmed progression and/or unacceptable toxicity. Among 35 patients, 29 had OPSCC. Moreover, of the total treated patients, 29 were evaluable for response (confirmed HPV-16/18+ disease; received both agents), including 23 patients with OPSCC. The population was heterogeneous for prior lines of therapy, platinum sensitivity, tumor PD-L1 expression, and extent of disease. The updated results of the trial (23) showed that HPV-16/18–specific T cells, evaluated by ELISpot, increased on treatment; 4/8 evaluable patients had a >2-fold increase in tumor-infiltrating lymphocytes (TILs) CD8+ cells. The primary efficacy endpoint, ORR, was not met, since the lower bound of the 95% CI for ORR did not exclude the null hypothesis of ORR ≤15% (ORR 27.6% [95% CI, 12.7–47.2; 4 CR, 4 PR, which were independent of PD-L1 tumor-cell expression]). However, the potential clinical benefit was encouraging, with 5/8 responding patients who had not progressed by the data cutoff. Median PFS was 3.5 months (95% CI, 1.9–9.0) and mOS was 29.2 months (15.2–inestimable). Two patients (5.7%) discontinued treatment for grade 3 AEs.

#### 2.1.4 GL-0810

GL-0810 is an HPV-16-specific polypeptide vaccine prepared with a granulocyte-macrophage colony-stimulating factor and a montanide adjuvant for subcutaneous administration. This vaccine was evaluated in the cohort 1 of the phase I dose escalation trial NCT00257738 (24), in which 9 male patients with heavily pre-treated HPV-16+ R/M HNSCC – 8 of them with OPSCC – were enrolled. Three dose levels (500, 1,000, and 1,500 μg) of GL-0810 were administered subcutaneously every 2 weeks for a total of 4 vaccinations. Patients were distributed equally at each dose level (3 patients each). Among those who received all 4 vaccinations, 4/5 (80 %) developed antigen-specific T-cell and antibody responses to the vaccine. Significant concordance between T-cell and antibody responses was observed (p<0.001), with T-cell responses preceding antibodies detection. All 9 patients (8 with OPSCC) developed PD as per RECIST 1.1. Median PFS was 80 days and mOS was 196 days. The study was closed prematurely due to poor accrual. All participants completed treatment by trial termination. Overall, the vaccine was well tolerated with predominantly local grade 1 toxicity.

#### 2.1.5 HB-201 and HB-202

HB-200 comprises an alternating sequence of 2 live-attenuated arenavirus vectors, HB-201 and HB-202, derived from the lymphocytic choriomeningitis virus (LCMV) and Pichinde virus, respectively. HB-200 vectors express a non-oncogenic HPV16 E6E7 fusion protein and induce E6 and E7-specific CD8 T-cell responses. In the non-randomized phase II part of the NCT04180215 trial (25), participants with HPV16+ PD-L1 CPS ≥1 R/M HNSCC were treated with HB-200 (intravenously every 3 weeks (q3w) for the first 5 doses, then every 6 weeks (q6w)) in combination with pembrolizumab (200 mg q3w or 400 mg q6w). Forty-two patients with HNSCC (41 with OPSCC, 98% males) were treated with HB-200 plus pembrolizumab in the first-line setting. Forty patients (95%) were ICI naive. Median follow-up was 5.6 months. Among 35 evaluable patients – those with ≥1 tumor response assessment – ORR was 43% (59% in patients with CPS ≥20). In 22/31 patients, HB-200 in association with pembrolizumab increased circulating HPV16-specific CD8+ T cells to >1% of all CD8+ T cells. The safety profile of the treatment was manageable. Five of the 7 patients with grade ≥3 AEs experienced transient cytopenia limited to cycle 1.

#### 2.1.6 PRGN-2009

The vaccine PRGN-2009 uses a gorilla adenoviral-vector targeting HPV-16/18 E6, E7 and HPV-16 E5. This vaccine was tested in combination with bintrafusp alfa – a bifunctional fusion protein targeting Transforming Growth Factor (TGF)-β and PD-L1 – in the arm 1B of the phase I non-randomized trial NCT04432597 (26) – which included 7/11 patients with OPSCC (while 3 patients had cervical cancer and 1 anal cancer). In this cohort the ORR was 20% for all patients (22% in ICI-resistant patients), and mOS was 24.6 months (95% CI 9.6-NR). Seven out of 10 (70%) and 7/8 (88%) evaluable patients had increased HPV16- and HPV18-specific T cells, respectively. Of note, HPV-specific T-cell responses were observed concurrently with vector-neutralizing antibodies during the vaccine treatment period. No patients with OPSCC were enrolled in the dose escalation arm 1A evaluating PRGN-2009 monotherapy.

#### 2.1.7 TG4001 (tipapkinogen sovacivec)

TG4001 is a highly attenuated vaccinia vector expressing HPV-16 E6/E7 non-oncogenic proteins and IL-2. This vaccine was studied in combination with avelumab in the phase Ib/II non-randomized trial NCT03260023 (27), which enrolled patients with advanced HPV16+ cancers. Ten out of 43 patients (24%) had OPSCC. This combination was safe and demonstrated antitumor activity (ORR 22%) in heavily pre-treated HPV16+ cancer patients.

#### 2.1.8 SQZ-PBMC-HPV and SQZ-eAPC-HPV

SQZ-PBMC-HPV is a therapeutic vaccine consisting of autologous peripheral blood mononuclear cells (PBMC) loaded by microfluidic squeezing with HPV16 E6 and E7 antigens. In the phase I dose-escalation part of the trial NCT04084951 (28), patients with human leukocyte antigen (HLA)-A*02+ and advanced/metastatic HPV-16+ cancers received SQZ-PBMC-HPV q3w. Five out of 18 enrolled patients (27.8%) had OPSCC. A *post hoc* analysis (29) subdivided participants by change in CD8+ T-cell density in tumor biopsy between screening and day 8 of cycle 2. Seventeen of the 18 patients with serially biopsied tumor tissue were evaluable for response. Six (33%) had an increase in CD8+ T-cell density in the tumor and had improved DCR (66.7% *vs* 16.7%) and mOS (606.5 days *vs* 170.0 days, p=0.0078). The NCT04084951 trial also includes a part 2 in which SQZ-PBMC-HPV is administered in combination with ICI.

The phase I/II study NCT05357898 [(30), **Table 1**] of the autologous vaccine SQZ-eAPC-HPV, as monotherapy and in combination with pembrolizumab, in patients with HPV16+ locally advanced or R/M solid tumors, was terminated due to corporate decision.

**Table 1:** Clinical trials on HPV therapeutic vaccines including patients with OPSCC.

Clinical trials for patients with HPV+ OPSCC							
#	ID	Phase	Status	Vaccine name	Platform	Disease	Stage of the disease
1	NCT04852328	II	Recruiting	CUE-101	Peptide	HPV-16+ OPSCC	Early (neoadjuvant setting)
2	NCT03669718	II	Active, not recruiting	ISA101b	Peptide	HPV-16+ PD-L1+ (CPS ≥1) OPSCC	Advanced
3	NCT04398524	II	Active, not recruiting	ISA101b	Peptide	HPV-16+ OPSCC	Advanced
4	NCT03258008	II	Terminated^a^	ISA101b	Peptide	HPV+ OPSCC	Advanced
5	NCT05232851	I/II	Active, not recruiting	PDS0101	Peptide	HPV+ OPSCC	Locally advanced
6	NCT06007092	I/IIa	Recruiting	CD40HVac	Subunit vaccine	HPV-16+ OPSCC	Disease-free
7	NCT05799144	II	Recruiting	pBI-11; TA-HPV	DNA; viral vector	HPV+ PD-L1+ (CPS ≥1) OPSCC	Advanced
8	NCT05996523	II	Recruiting	PRGN-2009	Viral vector	HPV+ OPSCC	Early (before definitive treatment)
9	NCT06223568	II	Recruiting	PRGN-2009	Viral vector	HPV+ OPSCC	Early (neoadjuvant setting)
10	NCT05108870	I/II	Recruiting	HB-200	Viral vector	HPV-16+ OPSCC	Early (neoadjuvant setting)
11	NCT02002182	II	Completed	ADXS11-001	Bacterial vector	HPV+ OPSCC	Early (neoadjuvant setting)
12	NCT01598792	I	Terminated^b^	ADXS11-001	Bacterial vector	HPV-16+ OPSCC	Disease-free
13	NCT06016920	I/IIa	Recruiting	VB10.16	DNA	HPV-16+ PD-L1+ (CPS ≥1) OPSCC	Advanced
14	NCT04001413	II	Withdrawn^c^	MEDI0457 (INO-3112)	DNA	HPV-16+ OPSCC	Early (MRD)

Clinical trials for patients with HPV+ HNSCC							
#	ID	Phase	Status	Vaccine name	Platform	Disease	Stage of the disease
15	NCT03978689	I	Active, not recruiting	CUE-101	Peptide	HPV-16+ HNSCC	Advanced
16	NCT04369937	II	Active, not recruiting	ISA101b	Peptide	HPV-16+ HNSCC	Locally advanced
17	NCT06790966	III	Recruiting	PDS0101	Peptide	HPV-16+ PD-L1+ (CPS ≥1) HNSCC	Advanced
18	NCT04260126	II	Active, not recruiting	PDS0101	Peptide	HPV-16+ PD-L1+ (CPS ≥1) HNSCC	Advanced
19	NCT00257738	I	Completed	GL-0810	Peptide	MAGE-A3+ or HPV-16+ HNSCC	Advanced
20	NCT06373380	I	Recruiting	HB-200	Viral vector	HPV-16+ HNSCC	Early (MRD)
21	NCT04534205	II/III	Recruiting	BNT113	RNA	HPV-16+ PD-L1+ (CPS ≥1) HNSCC	Advanced
22	NCT03162224	Ib/IIa	Completed	MEDI0457 (INO-3112)	DNA	HPV-16/18+ HNSCC	Advanced
23	NCT02163057	I/IIa	Completed	MEDI0457 (INO-3112)	DNA	HPV-16/18+ HNSCC	Early (before ± after definitive treatment)
24	NCT05286060	II	Recruiting	GX-188E	DNA	HPV-16 and/or 18+ HNSCC	Early (neoadjuvant setting)
25	NCT05280457	II	Recruiting	GX-188E	DNA	HPV-16 and/or 18+ HNSCC	Advanced
26	NCT01493154	I	Terminated^d^	pNGVL4a-CRT/E7 (Detox)	DNA	HPV-16+ HNSCC	Locally advanced/advanced

Clinical trials for patients with HPV+ cancers							
#	ID	Phase	Status	Vaccine name	Platform	Disease	Stage of the disease
27	NCT02426892	II	Completed	ISA101b	Peptide	HPV-16+ cancers	Advanced
28	NCT04287868	I/II	Active, not recruiting	PDS0101	Peptide	HPV+ cancers	Locally advanced/advanced
29	NCT02865135	Ib/II	Completed	DPX-E7	Peptide	HPV-16+ cancers	Advanced
30	NCT02821494	I	Completed	HESPeCTA	Peptide	HPV-16+ cancers or premalignant lesions	Disease-free or premalignant lesions
31	NCT04432597	I/II	Active, not recruiting	PRGN-2009	Viral vector	HPV+ cancers/OPSCC or SNSCC	Locally advanced/advanced
32	NCT04180215	I/II	Completed	HB-200	Viral vector	HPV-16+ cancers	Advanced
33	NCT03260023	Ib/II	Active, not recruiting	TG4001	Viral vector	HPV-16+ cancers	Advanced
34	NCT06319963	I/IIa	Recruiting	Lenti-HPV-07	Viral vector	HPV+ OPSCC or cervical cancer	Locally advanced/advanced
35	NCT02291055	I/II	Terminated^e^	ADXS11-001	Bacterial vector	HPV+ HNSCC or cervical cancer	Advanced
36	NCT03418480	I/II	Completed	BNT113	RNA	HPV-16+ cancers	Disease-free or advanced
37	NCT04084951	I	Completed	SQZ-PBMC-HPV	Autologous	HPV-16+ cancers	Advanced
38	NCT05357898	I/II	Terminated^f^	SQZ-eAPC-HPV	Autologous	HPV-16+ cancers	Locally advanced/advanced
39	NCT00019110	I	Completed	PBMC and HPV-16 E7/E6 peptides	Autologous; peptide	HPV-16+ cancers	Locally advanced/advanced

Other clinical trials							
#	ID	Phase	Status	Vaccine name	Platform	Disease	Stage of the disease
40	NCT03821272	I/II	Completed	PepCan	Peptide	HNSCC, regardless of HPV status	Disease-free

^a^ Due to slow accrual; ^b^ one patient suffered dose-limiting toxicity. Vaccine manufacturers withdrew support on commercial grounds; ^c^ funding withdrawn; ^d^ funding cessation; ^e^ early termination based on discussion with FDA (1-year surveillance period considered sufficient); ^f^ corporate decision.

APC, antigen-presenting cells; MRD, minimal residual disease; SNSCC, sinonasal squamous cell carcinoma The alternation between gray shaded and white lines visually highlights the clinical trials including patients with OPSCC related to the same therapeutic vaccine, which are grouped in clinical trials for patients with HPV+ OPSCC, HPV+ HNSCC, HPV+ cancers, other. In the column “Status”, the colored text highlights the status of the trials in terms of completion as of August 31, 2025: recruiting (green); active, not recruiting (blue); completed, terminated, withdrawn (black).

#### 2.1.9 PDS0101

PDS0101 is a liposomal multipeptide therapeutic vaccine targeting the HPV-16 E6 and E7 oncoproteins. In the phase I/II single-arm trial NCT04287868 (31), the combination of PDS0101, the tumor-targeting human IL-12 antibody-drug conjugate PDS01ADC, and bintrafusp alfa in 50 patients with advanced/metastatic HPV+ cancers, including 21 patients (42%) with OPSCC, showed an acceptable safety profile, promising antitumor activity, and improved OS. With a median follow-up of 37.7 (30.6-42.0) months, the ORR and the mOS were, respectively, 35.7% (95% CI, 12.8%-64.9%) and 42.4 months (95% CI, 8.3 months-inestimable) in the ICI naive subgroup, and 16.7% (95% CI, 6.4%-32.8%) and 15.8 months (95% CI, 9.0-21.3 months) in the ICI– resistant patients.

The combination PDS0101-pembrolizumab was studied as first-line treatment in patients with HPV-16+ R/M HNSCC with CPS ≥1, including OPSCC, in the study NCT04260126 VERSATILE-002 (32). With a median follow-up of 18.4 (0.2-42.7) months, this association therapy was well tolerated and demonstrated deep and durable clinical responses (ORR 35.8%, median duration 21.8 (11.5-inestimable) months) among the 53 enrolled patients.

These results led to the design of the randomized phase III trial NCT06790966 VERSATILE-003 (33), which is evaluating PDS0101-pembrolizumab *versus* pembrolizumab and has OS as the primary endpoint. The study is recruiting patients with HPV16+ R/M HNSCC, including OPSCC, with PD-L1+ disease (CPS ≥1).

#### 2.1.10 CUE-101

CUE-101 is a fusion protein composed of an HLA complex (HLA-A*0201), a peptide epitope derived from the HPV16 E7 protein and 4 molecules of attenuated IL-2. This vaccine was evaluated in a phase I dose-escalation and expansion trial (NCT03978689) (34), administered as monotherapy and in combination with pembrolizumab, in patients with R/M HNSCC, including OPSCC. Among the 19 evaluable patients treated with the combination therapy, the ORR was 47% and the mPFS was 5.8 months. Among the participants who receive CUE-101 monotherapy, a mOS of 20.8 months was observed.

Other therapeutic vaccines were evaluated in the R/M setting. As an example, the lipoplex-formulated mRNA therapeutic vaccine BNT113 was assessed in the arm 1B of the phase I/II, intra-patient dose escalation trial NCT03418480 (35), which enrolled participants with HPV16+ advanced cancers, including 3 patients with HNSCC. DPX-E7 (peptide-based vaccine), VB10.16 (DNA-based) and pNGVL4a-CRT/E7(Detox) (DNA-based) were also assessed in the advanced setting. Information on the related clinical trials is reported in **Table 1**.

### 2.2 Recruiting clinical trials

#### 2.2.1 BNT113

The HPV16 E6/E7 mRNA cancer vaccine BNT113 is also under evaluation in the study AHEAD-MERIT (NCT04534205) (36), a randomized phase II/III trial of BNT113 plus pembrolizumab *versus* pembrolizumab monotherapy as first-line treatment. The study is recruiting patients with unresectable R/M HPV16+ PD-L1+ (CPS ≥1) HNSCC, including patients with OPSCC. In the non-randomized safety run-in phase conducted in 15 male patients, the association therapy was well-tolerated and showed encouraging signs of efficacy. After 12 months of median follow-up, unconfirmed ORR was 40%, mPFS was 3.9 (95% CI: 2.1, 12.9) months, mOS was 22.6 (11.8-inestimable) months.

#### 2.2.2 pBI-11

pBI-11 is a DNA vaccine encoding mycobacteria heat shock protein 70 linked to HPV16/18 E6/E7 proteins. The ongoing phase II study NCT05799144 (37), with a 6 patient safety run-in phase, is evaluating pBI-11 in combination with pembrolizumab and TA-HPV, a recombinant vaccinia virus expressing modified HPV16/18 E6 and E7. The trial is open to recruit patients with R/M HPV+ OPSCC without prior therapy for R/M disease, and with a PD-L1 CPS >1. Blood and tissue will be tested to assess specific immune responses.

Other therapeutic vaccines, such as Lenti-HPV-07 (a viral vector-based vaccine) or GX-118 (a DNA-based vaccine), are being evaluated in advanced settings for patients with HPV+ tumors or HPV+ HNSCC, including OPSCC. Information on the related clinical trials is reported in **Table 1**.

## 3 HPV therapeutic vaccines for HPV+ OPSCC in the early setting

While HPV+ OPSCC in the early setting is highly responsive to chemoradiotherapy or surgery and with overall good outcomes, treatment frequently results in significant toxicity and functional sequelae. In this context, therapeutic vaccines could represent a strategy to further deepen responses and facilitate the de-escalation of standard therapy.

### 3.1 Completed or active but not recruiting clinical trials

#### 3.1.1 AMV002

The therapeutic HPV DNA vaccine AMV002 was evaluated in the phase I dose-escalation trial ACTRN12618000140257 (38). Eligible participants should have no evidence of R/M disease at least 12 weeks following the completion of curative treatment for their loco-regionally confined HPV+ OPSCC. AMV002 was administered intradermally 3 times every 4 weeks at 3 different dose levels (0.25, 1, and 4 mg/dose) in 3 sequential groups of 4 male patients each. All 12 participants completed the vaccination program and experienced mild discomfort at the injection site. After vaccination, 10/12 (83.3%) patients displayed a positive T-cell immune response to one or more of the E6 and/or E7 peptide pools by ELISpot assay. Moreover, vaccination resulted in a ≥4-fold increase in anti-HPV16 E7 antibody titer in one subject in the highest dose level group.

#### 3.1.2 MEDI0457 (INO-3112)

The HPV DNA vaccine MEDI0457 was evaluated in the prospective, 2-cohort, phase I/II trial, NCT02163057 (39), that recruited patients with HPV-16/-18+ HNSCC. All the 22 enrolled patients had locally advanced OPSCC, since the primary site of the cancer was the base of tongue (10 patients) or tonsil (12 patients). Patients were initially enrolled solely based on p16 positivity. HPV genotyping was performed retrospectively, and consequently 3 patients with non-HPV-16/-18+ tumors were enrolled. Patients in cohort I (n=6) received the vaccine before and after definitive surgery, while patients in cohort II (n=16) received MEDI0457 after the completion of concurrent chemoradiotherapy. No treatment-related grade 3–5 AEs occurred. MEDI0457 induced a strong, long-lived humoral response across both cohorts. Eighteen out of 21 evaluable patients showed elevated antigen-specific T-cell activity by IFN-γ ELISpot, and persistent cellular responses were noted up to 1 year. At the tissue level, the vaccine induced an alteration in the composition of TILs, with an increase in T CD8+ cells, denoting a proinflammatory response. At the median follow-up of 15.9 months, all patients were alive, 3 patients developed PD, and the 12-months DFS rate was 89.4%.

#### 3.1.3 PDS0101

The multipeptide vaccine PDS0101 was evaluated in the phase I/II trial MC200710 (NCT05232851) (40) in patients with HPV+ locally advanced OPSCC. Patients received 2 cycles of neoadjuvant PDS0101 alone (arm A) or in combination with pembrolizumab (arm B) prior to surgical resection or chemoradiotherapy. Twenty patients (10 per arm) were enrolled, and the demographics were homogeneous between the 2 arms (90% men, median age 61). The initial results, including primary endpoint of ctDNA response, have been presented. None of the 10 patients in arm A had a ≥50% decline in ctDNA from baseline, while 5/10 patients met this primary endpoint in arm B (p=0.03). After cycle 2, based on RECIST 1.1, 7 patients in arm A had SD; in arm B, 2 patients had PR and 8 had SD. With a median follow-up of 6 months, 2 patients in arm A recurred and none in arm B.

#### 3.1.4 ADX11-001

ADXS11–001 is a HPV16-E7 targeting vaccine, having as a vector a genetically modified, live, attenuated Listeria monocytogenes. A window of opportunity trial (NCT02002182) evaluated ADXS11–001 as a neoadjuvant vaccine in patients with newly diagnosed HPV+ OPSCC, prior to transoral robotic surgery (TORS). An interim analysis reported that 5/8 patients showed increased E6 or E7-specific IFN-γ responses post-vaccine and 4/8 patients demonstrated increased CD8+ and CD4+ TILs (41). While peripheral CD8+ cytotoxic T-cell levels remained constant during the study, there was a trend towards their increased expression of Lymphocyte-Activation Gene 3 (LAG-3) and PD-1–6 weeks post-surgery, consistent with activation. Moreover, 4/8 patients demonstrated expansion of specific T-cell receptors clones post-vaccination (42). It should be noted that 55.5% of the vaccinated patients had serious AEs (43). The study stopped recruitment after 15 participants.

#### 3.1.5 HESPeCTA

HESPeCTA is a therapeutic vaccine consisting of 2 SLPs derived from the HPV16 E6 oncoprotein and conjugated with amplivant, a synthetic toll-like receptor 2 ligand. Its safety and immunogenicity were evaluated in the phase I dose escalation NCT02821494 trial (44), initially designed to enroll patients with HPV+ OPSCC after curative-intent treatment, and then amended – due to the slow accrual – to enroll participants with different HPV+ tumors or pre-malignant lesions following standard curative treatment. The vaccine was injected intradermally 3 times q3w in 4 dose groups (1, 5, 20 or 50 μg per conjugated peptide). Twenty-five patients were enrolled, of which 12 patients with OPSCC (10 men and 2 women). In the lowest dose group (6/6 patients with OPSCC), vaccine-induced T-cell responses were observed in the blood of 3/6 vaccinated participants. All patients displayed a strong HPV16-specific T-cell response after vaccination in the highest dose group (including 2 patients with OPSCC). These HPV16-specific T-cell responses lasted until the end of the study. All patients with OPSCC had no evidence of disease at the latest follow-up.

Even in the early disease setting, some trials on HPV epitope-based therapeutic vaccines were open to recruit a broader population of patients with HPV+ HNSCC. As an example, arm 1A of the phase I/II non-randomized dose-escalation trial NCT03418480 [(35), **Table 1**] evaluating BNT113, which included 17 patients with resected HPV16+ HNSCC, disease-free at least 12 months after curative treatment.

### 3.2 Recruiting clinical trials

#### 3.2.1 HB-201 and HB-202

HB-200 viral vectors are being evaluated in the phase I/II TARGET-HPV trial (NCT05108870) (45) in patients with non-metastatic HPV-16+ OPSCC. Enrollment to the randomized phase II part is ongoing. In the phase I part, patients were treated with escalating doses of HB-201 single vector (arm A) or HB-202/201 alternating vector (arm B) for 3 times with neoadjuvant carboplatin on day 1 and paclitaxel on days 1, 8, 15 of q3w for 3 cycles. Deep responders (≥50% shrinkage) received TORS alone or with de-escalated radiotherapy ± chemotherapy, while non-responders received chemoradiotherapy. Twenty-one patients (median age 57, 91% men) were enrolled and treated (arm A, n=9; arm B, n=12). Grade ≥3 treatment-emergent AEs occurred in 13 (62%) patients overall. Deep responses following HB-200/chemotherapy were observed in 17/21 (81%) patients. All 3 patients who underwent TORS had no viable tumor at surgery. Two patients had persistent disease following chemoradiotherapy and underwent salvage surgery with no evidence of disease at the last follow-up.

#### 3.2.2 CUE-101

The ongoing non-randomized phase 2 trial NCT04852328 (46), is evaluating the fusion protein CUE-101, administered in 3 schedules (10 patients each) before curative-intent treatment to HLA-A*0201+ patients with HPV16+ OPSCC. Administration of CUE-101 as per schedule A (4 mg/kg intravenously 14 days) and B (14 and 7 days) before curative-intent treatment was safe and tolerable. Follow-up for patients who received schedules A and B is ongoing, while cohort C (schedule 7 days before curative treatment) is actively enrolling patients.

#### 3.2.3 PRGN-2009

The ongoing single-arm phase II trial NCT05996523 (47) is investigating the biological activity of neoadjuvant PRGN-2009 and pembrolizumab in patients with newly diagnosed stage I-III HPV+ OPSCC, who are candidates for definitive therapy (either surgery or chemoradiotherapy). The primary objective is to evaluate the antitumor immune response of this combination therapy (determined as a ≥2-fold increase in tumor-infiltrating CD3+ cells).

#### 3.2.4 CD40HVac

CD40HVac is a subunit vaccine composed of a humanized IgG4 monoclonal antibody fused to HPV16 E6/E7 proteins. The phase I/IIa dose-escalation trial NCT06007092 (48) planned to enroll patients with HPV16+ OPSCC, in complete remission after curative treatment. Twelve patients were included per dose group of, respectively, 1 mg (completed) or 3 mg (ongoing) CD40HVac subcutaneous adjuvanted with Hiltonol, and randomized 5:1 to receive vaccine or placebo at week 0, 4, and 24 starting 16–22 weeks after the end of curative treatment. In the first cohort of 12 patients (median age 56 years, 67% men) the treatment was well tolerated and induced, after 2 boosts at the lowest dose, specific and functional T-cell responses.

#### 3.2.5 GX-188E

Finally, it is worth mentioning GX-188E, a therapeutic DNA vaccine that encodes both E6 and E7. It is being evaluated in the neoadjuvant setting in association with pembrolizumab and the recombinant IL-7 GX-I7 in the phase II single-arm NCT05286060 trial (49) which is open to recruit patients with resectable HPV-16 and/or 18+ HNSCC, including OPSCC.

Other therapeutic vaccines such as PepCan (peptide-based vaccine) or pNGVL4a-CRT/E7(Detox) (DNA-based) were evaluated in the early setting, in patients with HNSCC, including OPSCC. Information on the related clinical trials is reported in **Table 1**.

## 4 Current limitations and challenges in clinical trials

Growing evidence and promising results led FDA to grant the Fast Track Designation to the combination PDS0101-Pembrolizumab in R/M HPV16+ HNSCC (50) and to ISA101b in R/M HPV-16+ OPSCC (20). However, the current landscape is characterized by heterogeneity in study population, investigational setting, vaccine platforms, combination therapy, and outcome measures. This variability limits the ability to compare studies and draw firm conclusions regarding the immunogenicity and clinical benefit of therapeutic vaccines. Furthermore, the frequent use of non-randomized, single-arm study designs and small sample sizes reduces the interpretability of individual trial results. Notably, financial interests in vaccine companies were frequently disclosed among the co-authors of the published studies.

### 4.1 Study design

Few clinical trials in different settings randomized patients with HPV+ OPSCC to receive a therapeutic vaccine – monotherapy or in combination with an ICI – *versus* placebo (15, 48). The majority of the studies, having a single-arm design, allowed for the assessment of immunogenicity, but made it difficult to isolate the vaccine’s true effects. Prospective trials enrolling multiple cohorts of patients, including, for instance, patients eligible for definitive surgery or patients who received chemoradiotherapy, were also conducted (39).

### 4.2 Study population and investigational setting

Few studies included a selected population of patients with HPV+ OPSCC, while more often patients with HNSCC from sites other than oropharynx or other HPV+ tumors were eligible to be enrolled. This may be linked to recruitment challenges, as in the phase I NCT02821494 trial (44).

Moreover, likely due to the limited sample size, subgroup analyses were not performed, and data were often not stratified according to the primary tumor site.

In the R/M setting, the enrolled study population was markedly heterogeneous in terms of PD-L1 status, prior systemic therapy, and platinum refractoriness, which may have had an impact on the observed efficacy of the vaccine in combinations with ICI. In fact, the inclusion of heavily pretreated patients, in which immune response to vaccination takes longer to develop and may not even develop at all, might have reduced its potential to slow disease progression. Even in earlier disease settings, heterogeneity was evident in terms of ongoing or prior treatments.

In both clinical practice and trial settings, p16 immunohistochemistry is the most extensively used biomarker assay for inferring HPV causation in OPSCC, due to challenges of direct HPV testing compared to the feasibility and the low cost of immunoistochemistry. However, discordance between p16 and HPV DNA or RNA status exists, and up to 20% of patients who have p16-positive tumors test negative for HPV DNA or RNA (51). For this reason, in some studies, bias may have been introduced in selecting patients using p16 as a surrogate marker of HPV-positivity, instead of HPV genotyping. As an example, in the NCT02163057 trial (39), patients were initially not selected based on HPV genotyping but were enrolled solely on the basis of p16 positivity. HPV genotyping was performed retrospectively, and consequently, 3 patients with non-HPV16 and 18 tumors were enrolled. Subsequently, the protocol was amended to require demonstration of HPV16 and/or HPV18 positivity. Some studies (21, 23, 38) allowed to enroll patients based on assessment of either p16 or nucleic acid testing. For instance, in the NCT03162224 trial (23), local assessment of either p16 or nucleic acid testing was used to determine HPV status for patient eligibility. HPV status was confirmed by central laboratory nucleic acid testing, and of the 30 patients with p16-positive tumors, 25 had HPV-16/18+ cancers on central laboratory nucleic acid testing. For this reason, in the future, combining p16 overexpression and HPV genotype assessment is essential.

Moreover, the proper selection of patients who could achieve the highest ORR and benefit to vaccination is crucial to obtain implementable results. Therefore, it is necessary that rigorous translational research is carried out so that an enriched population might be selected for further trials.

Finally, it should be highlighted that women are often underrepresented, as well as older cancer patients, and the sample size is small – with few exceptions (15, 17) – impacting the potential representativeness of the population.

### 4.3 Vaccines

Therapeutic vaccines targeting HPV epitopes administered to patients with OPSCC are heterogeneous in terms of platforms, adjuvants, method of administration, and combination treatment.

The platforms of interest include the peptide-based cancer vaccine ISA101b (15–19), a DNA-based vaccine such as MEDI0457 (INO-3112) (39, 52), and the live attenuated vectors HB-201 and HB-202 (45).

The method of administration varies according to the type of vaccines. For instance, MEDI0457 is administered by intramuscular injection followed immediately by electroporation (39); AMV002 is delivered intradermally (21, 38), while HB-201 and HB-202 are administered intravenously (45).

Moreover, differences could be found in vaccine adjuvants, which are known for enhancing the therapeutic efficacy of vaccination (53). They could range from immunostimulants such as IL-12 (52) or Hiltonol (48) to delivery systems such as plasmids incorporating a secretory sequence to induce helper T-cell responses, or a ubiquitin sequence, facilitating antigen processing and induction of cytotoxic T cells (21, 38), or IL-2 encoding plasmids (39).

Finally, the vaccine could be administered as a monotherapy, more often in the context of a phase I dose-escalation trial (38) or in combination with other systemic treatments, in particular ICI such as durvalumab (21) or cemiplimab (15–17), but also chemotherapy (45).

In a combination strategy, a cancer vaccine may overcome the resistance of certain tumors to ICI, while ICI may enhance the efficacy of the therapeutic vaccine (54) (**Figure 1**). Whilst this synergic action may induce more effective antitumor immune responses, at the same time, this impedes the evaluation of the real efficacy of the vaccine and the making of comparisons among the vaccines.

Therefore, different types of vaccines could have elicited a different type of adaptive response, more oriented towards a T-cell response (45) or including also a humoral response (39), even considering the heterogeneity of adjuvants and combination therapy.

### 4.4 Outcomes measures

The ability of a therapeutic vaccine to turn a “cold” (immune-desert) tumor into an “hot” (immune-inflamed) one (55) was evaluated differently among the studies. While some trials focused on T-cells and humoral response in peripheral blood (38), other trials also performed a tissue correlative analysis (39). In some cases, differential gene expression analysis was performed to compare responders *versus* non-responders (19). The T-cell response was frequently measured using the IFN-γ ELISpot assay, although the time points may differ.

ORR was the most common primary efficacy endpoint in the majority of the trials; however, only in a few cases, iRECIST was used to assess response (21) instead of RECIST v1.1. Moreover, the evaluation of circulating HPV-DNA has been rarely investigated as a possible surrogate method of activity.

Survival outcomes were more frequently among the secondary endpoints.

Finally, it is worth noting that the quality-of-life assessment was underreported.

### 4.5 Conflict of interests

Financial interests in vaccine companies were frequently disclosed among the co-authors of the published studies. Whilst this can represent a risk of bias, at the same time, the presence of multiple interests can also be seen as a marker of collaboration between entities (56). As long as the output is not controlled by any one party, cross-sectoral collaborations often lead to robust science and timely drug development.

## 5 Conclusion and future perspectives

HPV therapeutic vaccines in OPSCC represent an attractive field, which has been developing rapidly over the last years. These vaccines strategies (DNA, mRNA, peptides) targeting E6/E7 oncoproteins showed manageable toxicities and encouraging results in phase I/II clinical trials. They have proven to be effective in inducing a *de novo*, antigen-specific T-cell response and potentially improving clinical outcomes, especially in the R/M setting and in combination with other standard treatments, such as ICI or chemoradiotherapy. For this reason, some of these strategies are nearing clinical application, and among them, the peptide-based vaccines ISA101b and PDS0101 are the most promising candidates. As for the early setting, therapeutic vaccines in patients with HPV+ OPSCC can facilitate the de-escalation of standard therapy while ensuring deepen responses. Beside vaccine platforms, novel methods of administration – such as intramuscular injection followed immediately by electroporation – have been explored and showed promising efficacy.

On the other hand, different challenges are recognized in this field. For instance, the issue of patients’ selection remains crucial, and clinical research should proceed with the design of trials in enriched populations of patients.

Additionally, despite the prevalence of HPV+ OPSCC is increasing for all sex (7), women remain underrepresented in clinical trials. A meta-analysis (57) on 20 randomized controlled trials of ICI – including two studies enrolling also patients with OPSCC – showed that the benefit of ICI is sex-dependent. It would be interesting to understand if this also applies to HPV therapeutic vaccines. For this reason, future research should ensure greater inclusion of women in clinical trials, thus allowing to perform sub-analyses on the efficacy of vaccine strategies in this population.

While most HPV immune therapies currently in clinical testing target the HPV oncoproteins E6 and E7, there is a need to include more HPV antigens in new therapeutic vaccines to increase their efficacy. In this regard, the oncoproteins E1, E2, and E5 could be promising novel targets (58, 59).

In the future, to determine the real benefit of therapeutic vaccines in HPV+ OPSCC compared to other HPV+ solid tumors, the design of dedicated trials enrolling only this selected population should be favored.
